# Central Odontogenic Fibroma: A Case Report

**DOI:** 10.7759/cureus.2556

**Published:** 2018-04-30

**Authors:** Mehr Zia, Areej Arshad, Zoya Zaheer

**Affiliations:** 1 Armed Forces Institute of Dentistry, CMH Lahore Medical College and Institute of Dentistry, Lahore, PAK

**Keywords:** odontogenic tumors, central odontogenic fibroma, oral tumours, oral pathology, oral surgery, oral radiology

## Abstract

A central odontogenic fibroma (COF) is a rare tumor of odontogenic origin with a diverse histopathology found in both the mandible and the maxilla. It can often be difficult to diagnose; therefore, it is necessary to evaluate the clinical, radiographic, and histopathological analyses of COF and co-relate them in a manner to make definitive diagnosis easier for the treating physician. Herein, we report and discuss the first known case of central odontogenic fibroma in Pakistan: a 16-year-old boy presenting as a hard bony painless swelling of the left mandibular region. It appeared as a large, well-defined unilocular radiolucency on the orthopantomogram, making it indistinguishable from other radiolucent tumors of the mandible. Histologically, the lesion consisted of nests of odontogenic epithelium in between the fibroblastic stroma, confirming a definitive diagnosis of COF. It was treated by conservative surgical excision followed by curettage and no postoperative complications were reported.

## Introduction

A central odontogenic fibroma (COF) is a rare and benign tumor accounting for only 0.1% of all odontogenic tumors [1-3]. It may evolve from the dental follicle, the dental papilla, or the periodontal membrane [4]. The lesion appears both in the maxilla and mandible; in the maxilla, it appears in the anterior region whereas, in the mandible, it involves the premolar and molar area [1].

On clinical examination, it usually presents as a non-tender swelling showing cortical expansion [3,5-6]. However, root displacement and root resorption have also been reported in cases where lesions are more severe [3].

Radiographically, the COF may be multilocular or unilocular with distinct borders although, in some rare cases, a mixed radiolucent and radiopaque appearance with undefined borders has also been observed [1].

A histological examination of the lesion reveals mature collagen interspersed with fibroblasts. The collagenous tissue is moderately dense to dense and nests of inactive odontogenic epithelium in strands may also be present [4]. Because of its varying histological features, the COF is often confused with other entities, such as desmoplastic fibromas and odontogenic myxomas. Therefore, it is important to correlate the clinical, radiographic, and histopathological features of this tumor to reach a definitive diagnosis [1,6-8].

In this case report, we describe a case of COF in a 16-year-old male, occurring in the left posterior mandibular region. We also highlight the clinical and histological features of this rarely occurring odontogenic tumor.

## Case presentation

A 16-year-old boy reported to the clinical department of Armed Forces Institute of Dentistry (AFID), Rawalpindi, Pakistan, with a slow-growing swelling on the left side of his face for the past two years. He had no other active complaints apart from a slight discomfort upon mastication. His past medical, family, and social history were considered non-contributory to the case.

**Figure 1:**
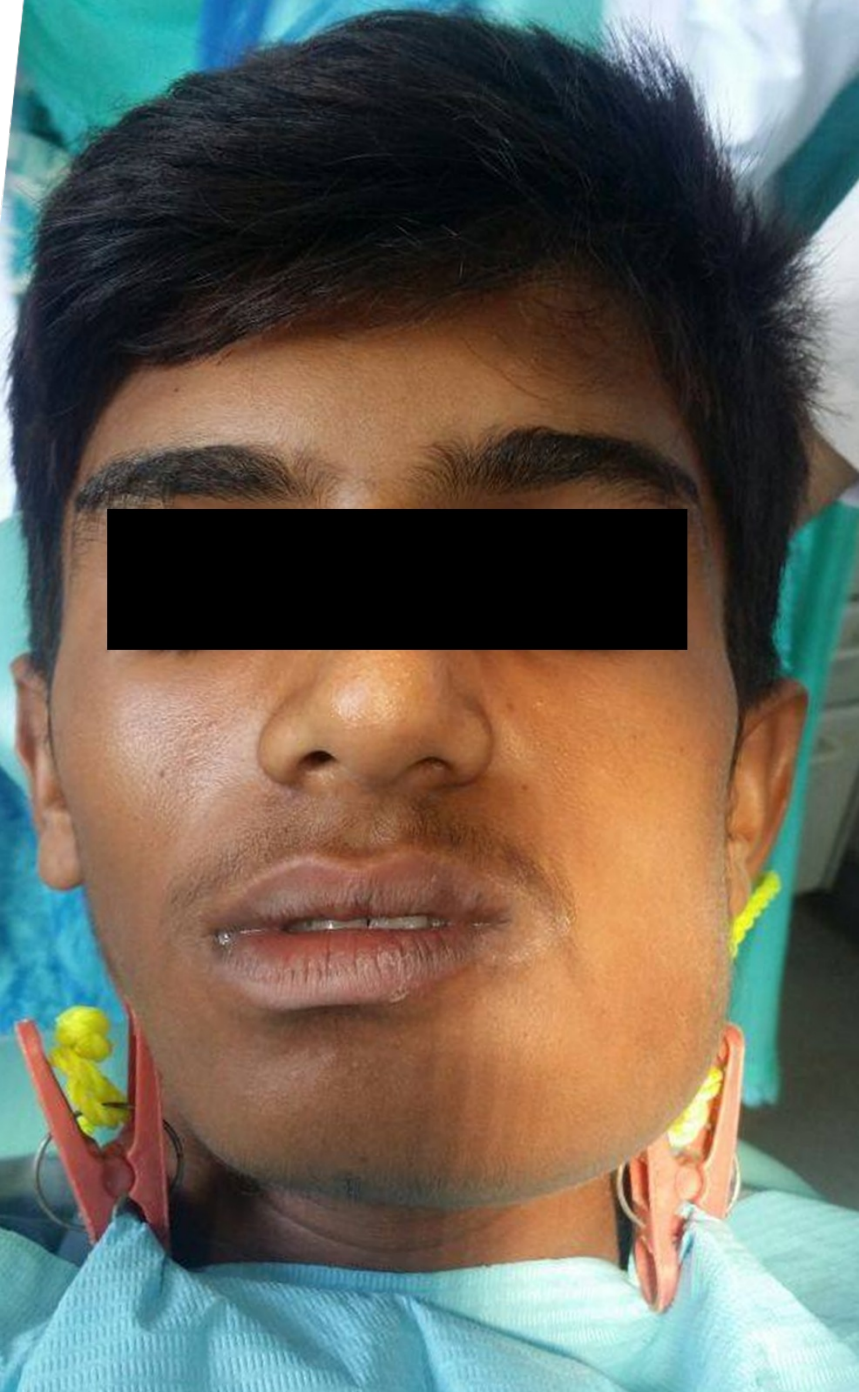
Initial presentation of the patient

Upon extraoral examination, marked facial asymmetry extending from the left parasymphysis to the angle of the mandible on the left side of the face was noticed, but no ulceration or change of color of the overlying skin was observed. The submandibular lymph node of the left side was palpable (Figure 1).

An intra-oral inspection revealed a swelling extending from tooth 32 to the ramus of the mandible (involving the retromolar area of the left side), causing a displacement of teeth 34, 35, and 37. A bicortical expansion of the mandibular plates was noticed, but there was no evidence of paresthesia.

**Figure 2:**
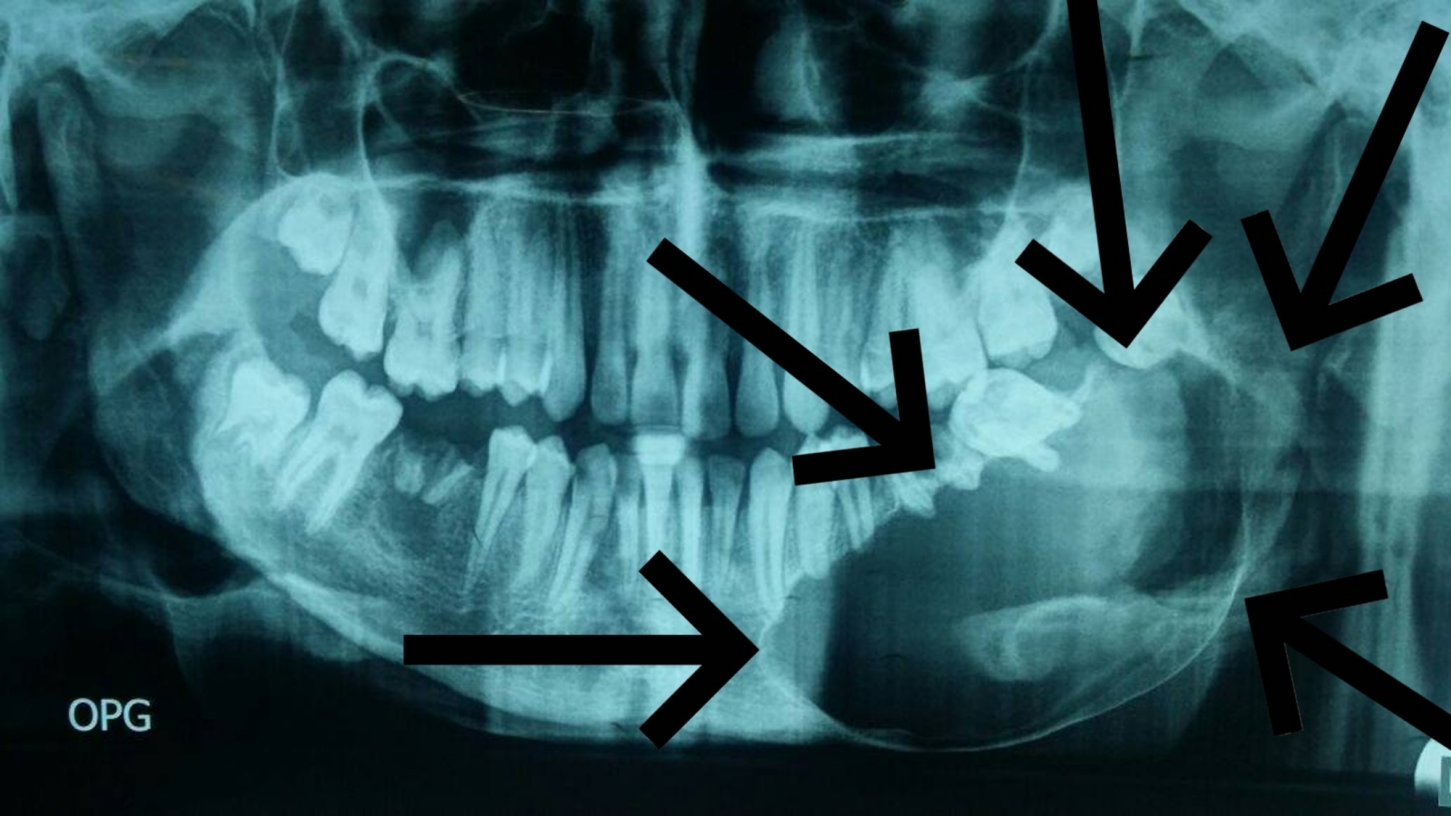
Orthopantomogram showing large well-defined radiolucency on the left side of the mandible

An orthopantomogram of the patient showed a large unilocular radiolucency extending from the left parasymphyseal area of the mandible up to the ramus; the inferior alveolar canal was displaced toward the lower border of the angle of the mandible. Root resorption of teeth 33, 34, 35, and 37 was seen along with the residual roots of tooth 36. The margins of the lesion appeared well defined (Figure 2).

Considering the clinical and radiographic evidence, a differential diagnosis of an odontogenic keratocystic tumor, unilocular ameloblastoma, and odontogenic myxoma was made.

An incisional biopsy revealed a fibro-osseous lesion and the section of the soft tissue showed fragments of fibrocollagenous tissue with a mild lymphoplasmacytic infiltrate. Sections from the bony tissue showed trabeculae of lamellar and woven bone with osteoblastic rimming and intervening spindle cell stroma.

**Figure 3:**
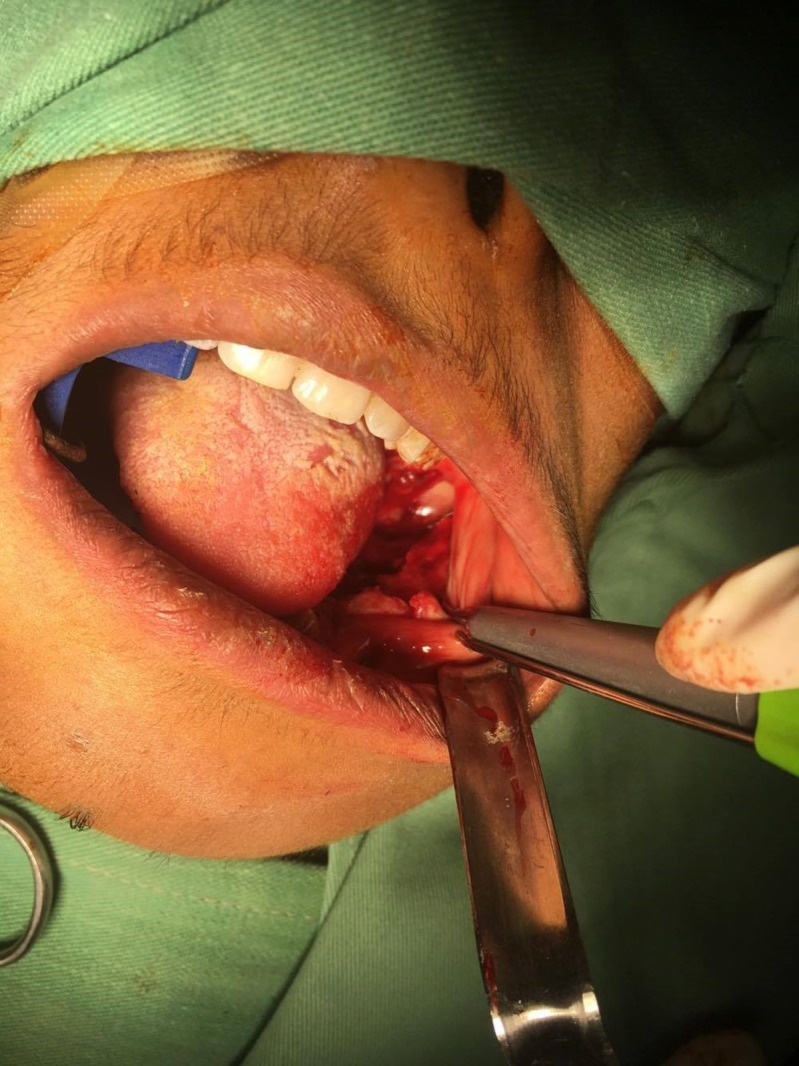
Clinical picture showing the exposure of the lesion through the extraction sockets

**Figure 4:**
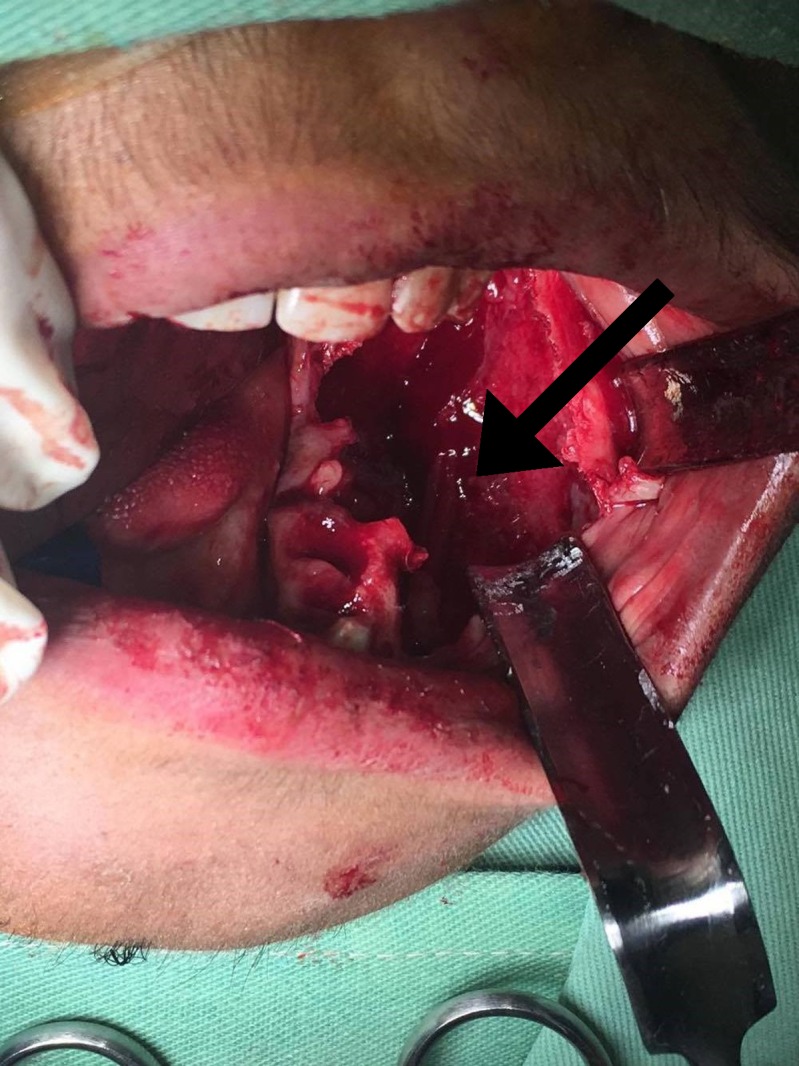
Arrow pointing towards the displaced inferior alveolar nerve (IAN)

Surgical excision of the lesion was planned two months after the biopsy. The lesion, along with spongy bone, was removed after being exposed intra-orally through the extraction site of teeth 34, 35, 36, and 37 (Figures 3-4).

**Figure 5:**
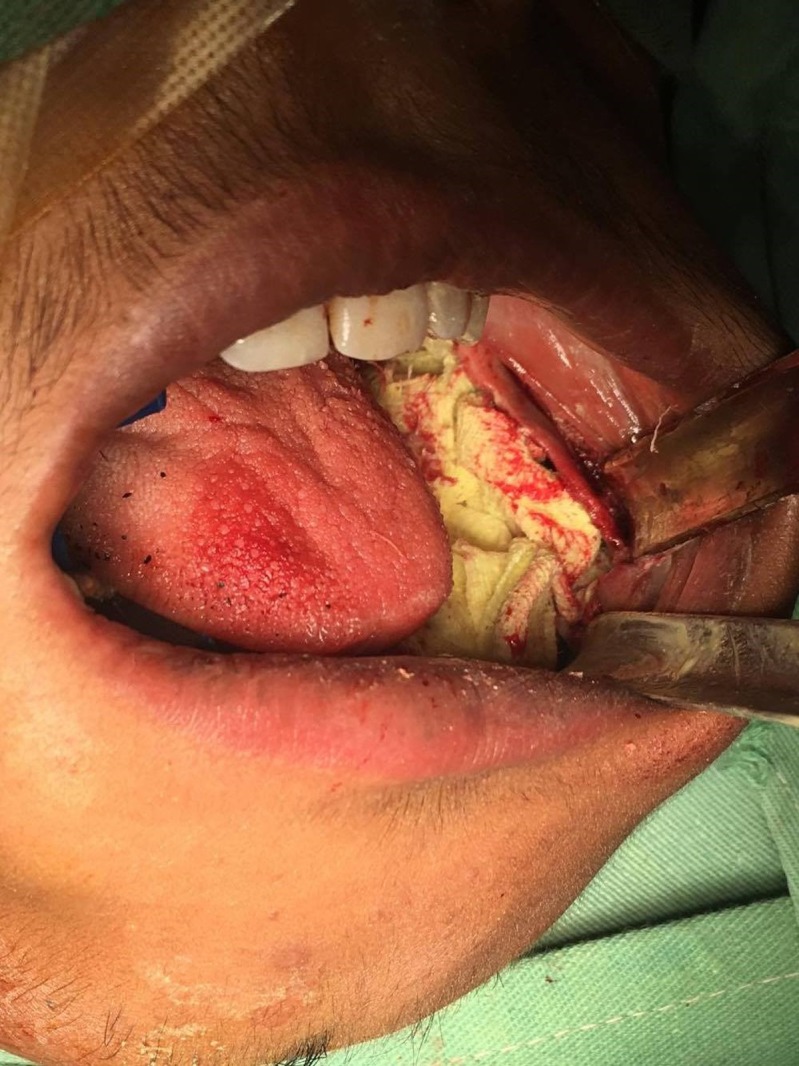
Final bismuth iodine paraffin paste (BIPP) dressing

After washing the cavity with normal saline, Cornoy’s solution was applied, taking special care of the inferior alveolar nerve. A bismuth iodine paraffin paste (BIPP) dressing was applied (Figure 5) and the specimen was sent for histopathological evaluation to the Armed Forces Institute of Pathology.

**Figure 6:**
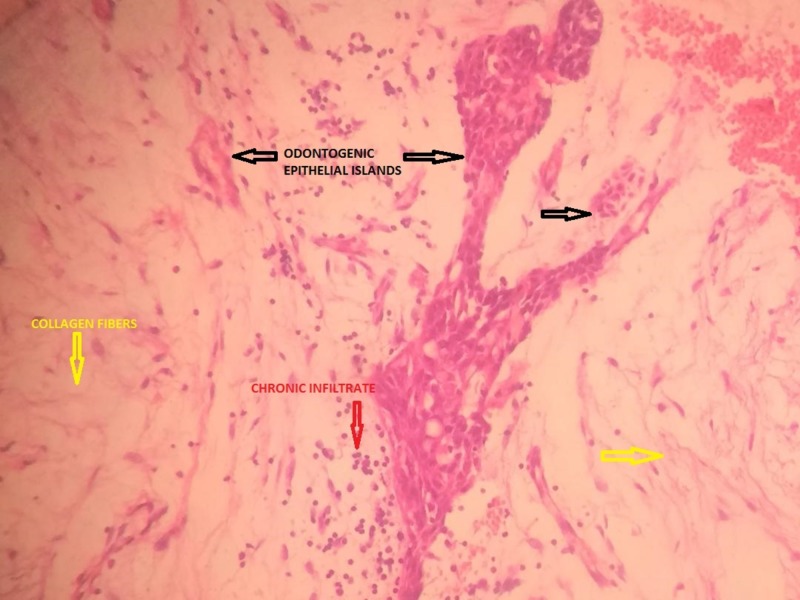
Section from the histopathological report showing nests of odontogenic epithelium in between fibroblastic stroma Black Arrows: Odontogenic Epithelial Islands; Yellow Arrows: Collagen Fibers; Red Arrow: Chronic Infiltrate.

The histopathological report concluded the presence of tissue fragments lined by stratified squamous epithelium with dense lymphoplasmacytic infiltrate. Other fragments from the specimen revealed loose spindle-shaped cells in fibrocollagenous stroma with a myxoid appearance in some areas. Moreover, nests of odontogenic epithelium were also seen in between the fibroblastic stroma (Figure 6).

**Figure 7:**
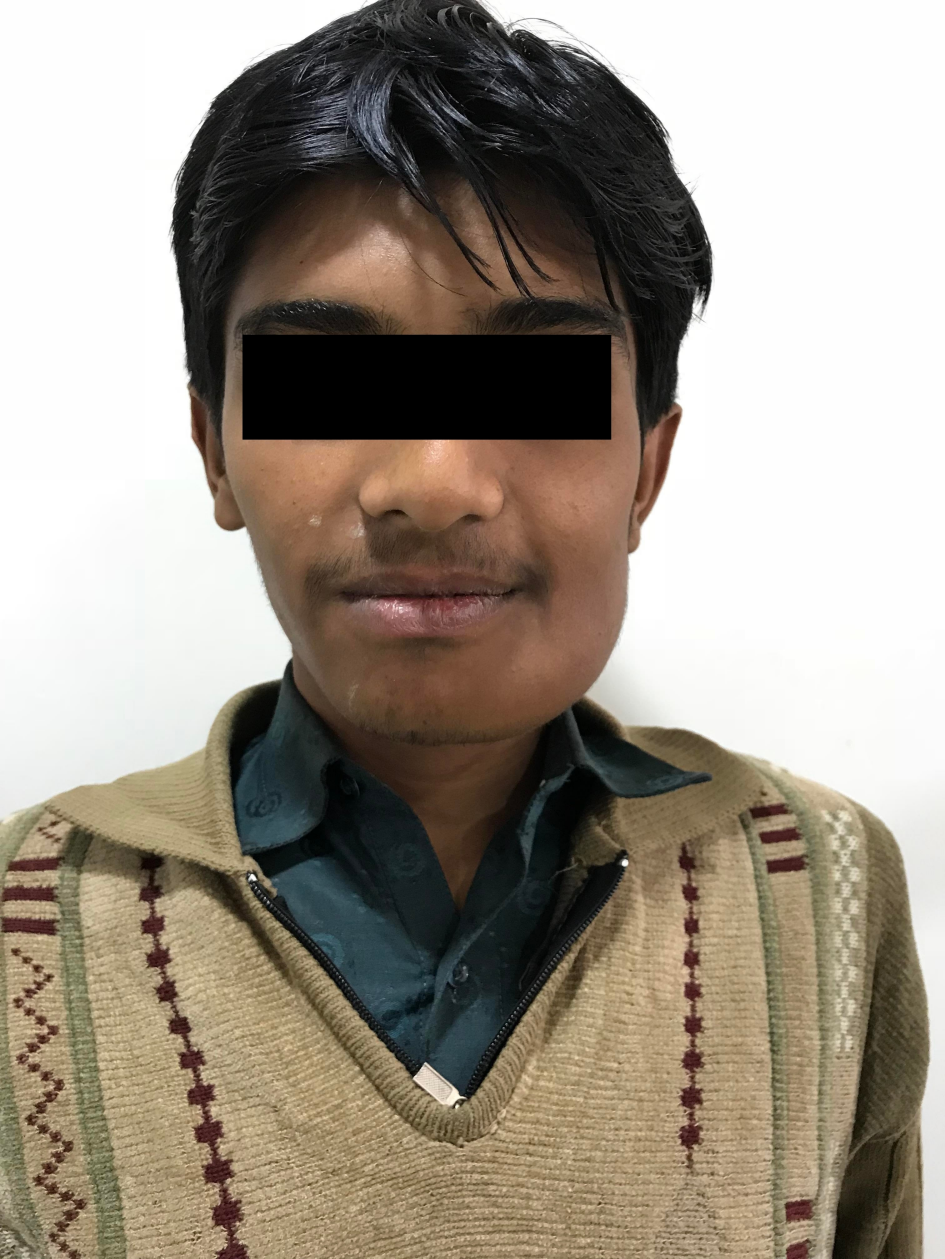
Significant decrease in the extraoral swelling on the third-month follow-up

Complementing previous findings with conclusive histopathological results, a final diagnosis of central odontogenic fibroma was established. The patient was scheduled for further follow-ups; on the third-month follow-up, a significant decrease in the patients extraoral swelling was observed (Figure 7).

## Discussion

COF can appear to be similar to other odontogenic tumors [8]. However, it is differentiated from such tumors based on its distinct clinical and histopathological features [9]; therefore, it is essential to distinguish COF from the other odontogenic tumors when attempting to diagnose it. The complete pathogenesis of COF is still not clear: whether it is a peculiar reactive process (hamartoma) or a neoplasm has not been completely established yet in the literature [2].

The occurrence of COF has been reported to be more common in females than males [2] and is usually diagnosed in patients in the second or third decades of life. Regardless, cases of patients between the ages of five and 80 years have also been reported [10]. Originally, the lesion was believed to be exclusively confined to the mandible but observations have shown it to occur with almost equal distribution in the maxilla (54.4%) and the mandible (45.6%) [7]. In this case, the patient was a 16-year-old male with the lesion occurring in the posterior mandible.

The most common clinical sign is the swelling of the maxilla or mandible while pain and paraesthesia are less frequently observed [3]. Moreover, COF is reported to have a slow growth rate, resulting in painless cortical expansion [1-2,5-6]. Consistent with these reports, our patient only complained of a painless slow-growing swelling with no associated paraesthesia.

Daniels et al. reported that radiographically, smaller-sized lesions often appeared unilocular whereas the larger lesions, exhibiting more aggressive behavior, such as root resorption of adjacent teeth and expansion of the cortical plates, were often multilocular [2]. In our case, however, the lesion, although large, aggressive, and causing the displacement of the inferior alveolar nerve (IAN), as well as the displacement of adjacent teeth, appeared as a unilocular radiolucency with well-defined borders on the radiograph, a finding not consistent with Daniels’ observation.

Histopathologically, COF has been categorized into two types: an epithelium-poor (simple type) and an epithelium-rich type, also referred to as complex or World Health Organisation (WHO) type [5]. The simple type has delicate fibers interposed with substantial amounts of ground substance and is comparatively acellular. The presence of small strands of inactive epithelium is a variable feature [3] but was observed in the histopathological report of our particular case.

Sections from the specimen of our patient showed fragments of tissue lined by stratified squamous epithelium with loose spindle-shaped cells in a fibrocollagenous stroma. It exhibited a myxoid appearance along with nests of odontogenic epithelium. These findings from our biopsy report allowed us to reach a definitive diagnosis of central odontogenic fibroma of the complex/WHO type.

It is important to include other benign tumors in the differential diagnosis of COF because odontogenic myxomas and enlarged, contiguous dental follicles may also show relatively uniform stellate or spindle-shaped cells embedded in a fibrocollagenous matrix [7]. Furthermore, the differential diagnosis of COF should include a wide range of fibrous tissue tumors, especially fibromyxoma and desmoplastic fibroma. Such distinctions are vital because these two lesions are associated with a significant recurrent rate, whereas COF is not [6].

The current recommended treatment for COF is conservative surgical enucleation followed by the curettage of the cavity. The recurrence rate is not common and a good prognosis has been witnessed in most cases [1-3,5-7].

## Conclusions

We report the first known case of COF, an uncommonly occurring odontogenic tumor, in Pakistan. This tumor is difficult to diagnose because of its non-specific clinical signs and symptoms. It is also generally confused with other tumors of odontogenic origin, as was seen in our case, which was clinically diagnosed as an odontogenic keratocystic tumor, leading to a hindrance in diagnosis and management. Apart from its histopathological classification into simple and complex/WHO types, there are no clear clinical or radiographic diagnostic guidelines to reach a definitive diagnosis of COF. Complete enucleation and curettage of the lesion seem to be the treatment of choice with a good prognosis and few chances of recurrence have been seen so far.
